# 超高效液相色谱-串联质谱同时测定尿液中10种邻苯二甲酸酯代谢物

**DOI:** 10.3724/SP.J.1123.2024.04002

**Published:** 2025-06-08

**Authors:** Xiaoying ZHAO, Hui KANG, Yanyan WANG, Wenhui LI, Ming CHEN, Chan KE, Feng QIN, Xixiong KANG

**Affiliations:** 1.沈阳药科大学分析化学实验室，辽宁 沈阳 110000; 1. Analytical Chemistry Laboratory，Shenyang Pharmaceutical University，Shenyang 110000，China; 2.北京中检体外诊断工程技术研究中心，北京 100095; 2. Beijing Zhongjian IVD Engineering Research Center，Beijing 100095，China; 3.裕菁科技（上海）有限公司，上海 200120; 3. Yujing Technology Shanghai Co. ，Ltd. ，Shanghai 200120，China

**Keywords:** 超高效液相色谱-串联质谱, 邻苯二甲酸酯代谢物, 尿液样品, 塑化剂, ultra performance liquid chromatography-tandem mass spectrometry （UPLC-MS/MS）, phthalate metabolites, urine samples, plasticizer

## Abstract

建立了超高效液相色谱-串联质谱（UPLC-MS/MS）同时测定人尿液中10种邻苯二甲酸酯（PAEs）代谢物的方法，并将该方法应用于人体内PAEs代谢物的暴露水平评估。采用固相萃取技术对尿液中的10种PAEs代谢物进行前处理，使用ACQUITY UPLC BEH Phenyl色谱柱（50 mm×2.1 mm， 1.7 μm）分离，以0.1%甲酸水和0.1%甲酸乙腈为流动相进行梯度洗脱，流速为0.5 mL/min，柱温为40 ℃，进样量为20 μL。在电喷雾电离（ESI）源负离子模式下进行检测，多反应监测模式采集数据，同位素内标法定量。方法学验证结果表明，该方法的专属性高，10种PAEs代谢物在各自的线性范围内线性关系良好，检出限（LOD）为0.03~0.3 ng/mL，定量限（LOQ）为0.1~1 ng/mL；在4个质控水平下，10种PAEs代谢物的日内和日间精密度均≤8.3%，准确度（用相对误差表示）为‒10.5%~7.3%。将该方法应用于评估60名志愿者尿液中PAEs代谢物的暴露水平，每名志愿者的尿液中均可检出1~6种PAEs代谢物。该方法灵敏准确，简便高效，适用于PAEs代谢物的大规模生物监测。

## Abstract (en)

Phthalates （PAEs） are widely employed as plasticizers in plastic products that are used in industrial， agricultural， food， medical， and other fields. PAEs are relatively weakly bonded to plastic products through non-covalent interactions. Consequently， PAEs can easily leak from the product into the environment， which exposes the public to PAEs through food intake， skin absorption from personal care products， and by inhaling air. Related studies have shown that PAEs are endocrine-disrupting substances and that long-term exposure to PAEs may result in diseases of the nervous， reproductive， cardiovascular and immune systems. In addition， excessive exposure to PAEs may trigger inflammatory responses and induce tumors. Therefore， establishing a highly sensitive assay for determining PAE levels in the human body following exposure is an important objective. PAEs generally have half-lives of less than 24 h； they are rapidly metabolized through enzymatic hydrolysis after entering the human body and excreted through urine. Therefore， most studies have focused on PAE metabolites as target compounds； hence， human body exposure to PAEs can be assessed by analyzing the types and levels of these metabolites. Herein， we established a method for simultaneously determining ten phthalate （PAE） metabolites in human urine using ultra performance liquid chromatography-tandem mass spectrometry （UPLC-MS/MS）. The ten PAE metabolites in urine were separated using an ACQUITY UPLC BEH Phenyl column （50 mm×2.1 mm， 1.7 μm）. Gradient elution was performed using 0.1% formic acid aqueous solution and 0.1% formic acid in acetonitrile as the mobile phases， at a flow rate of 0.5 mL/min， a column temperature of 40 ℃， and a sample size of 20 μL. Data were acquired in negative-ion electrospray ionization （ESI） and multiple reaction monitoring （MRM） modes， and quantified using the isotope internal standard method. The method was found to be highly specific， with the ten PAE metabolites exhibiting good linearities in their linear ranges， with limits of detection （LODs） and quantification （LOQs） of 0.03–0.3 and 0.1–1 ng/mL， respectively. Under the four quality control （QC） levels， the intra-day and inter-day precisions of the ten PAE metabolites were all ≤8.3%， and the accuracy ranged from ‒10.5% to 7.3%. The method was used to assess the exposure levels of PAE metabolites in the urine samples of 60 volunteers， with 1‒6 kinds of PAE metabolites detected in the urine of each volunteer. This method is sensitive， accurate， simple， efficient， and suitable for the large-scale biological monitoring of PAE metabolites.

邻苯二甲酸酯（phthalates，PAEs）是塑料制品中普遍使用的塑化剂，它们广泛存在于工业、农业、食品、医药等多个领域的产品中，如塑料地板、塑料薄膜、食品包装、血袋、玩具、化妆品和口罩等^［1‒3］^。PAEs与塑料制品之间通过非共价键结合，这种相互作用相对较弱，因此PAEs容易从产品中游离出来进入环境^［4］^。人群主要通过食物摄入^［5］^、个人护理产品的皮肤吸收^［6］^以及空气吸入^［7］^等途径暴露于PAEs。相关研究表明，PAEs是一种内分泌干扰物质，长期暴露于PAEs可能引发神经系统、生殖系统、心血管系统和免疫系统的疾病^［8］^。儿童通常是PAEs的高暴露风险群体^［9］^，性早熟、注意力缺陷多动障碍以及肥胖等问题与他们的PAEs暴露密切相关^［10］^。流行病学研究还指出，PAEs暴露与男性生殖器官异常、女性不孕以及子宫内膜异位症等疾病之间存在正相关关系^［11，12］^。此外，过度接触PAEs还可能引发炎症反应，甚至诱发肿瘤^［13，14］^。尽管塑化剂的使用受到法规限制，但其使用量仍然相当可观。在大气、室内灰尘、水源以及土壤中均可检测到高水平的PAEs^［15‒18］^。自20世纪90年代以来，美国、德国、加拿大等国家已将PAEs列为重点监测的有害化合物之一，并实施了全面且长期的生物监测计划^［19，20］^。相比之下，国内的相关报道较少，直到2017年才出现具有全国代表性的前瞻性队列研究^［21］^。

目前，相较于血浆、乳液、精液、羊水和唾液等生物基质，尿液是研究PAEs暴露的首选基质。尿液的非侵入性采集方式简便、快捷，且便于运输，这种无创采样方式也更易于受试者接受。然而，PAEs的半衰期通常不超过24 h，PAEs在进入人体后可迅速通过酶水解作用进行代谢，并通过尿液排出，这导致其在尿液中的含量低于其代谢产物的含量。此外，若将PAEs本身作为目标化合物进行研究，在样品运输和实验操作过程中，很难避免由于接触塑料尿杯、离心管等容器而引入的污染。因此，当前研究大多以PAEs代谢物为目标化合物，这些代谢产物与PAEs在人体内的代谢过程紧密相关。通过分析尿液中PAEs代谢物的种类和水平，可以评估人体对PAEs的暴露程度。

为准确测定PAEs代谢物的含量，建立高效、可靠的分析方法是关键前提。为了减少生物基质干扰，需在测定前进行样品前处理操作。当前，液液萃取和固相萃取是主要的PAEs代谢物前处理方法。液液萃取的成本虽低，但有机试剂用量大、味道刺激，且易导致样品乳化、净化效果差，需额外的净化步骤。相比之下，固相萃取方法具有回收率高、净化效果好、有机试剂消耗少及操作简便等优势。常见的固相萃取柱有C_18_固相萃取柱、亲水-亲酯平衡（hydrophile-lipophile balance，HLB）柱、混合型阴离子交换（mixed-mode anionic-exchanger，MAX）固相萃取柱等。MAX固相萃取柱兼具强阴离子交换和反相保留功能，可同时有效净化极性较强的单酯代谢物和极性较弱的长链次级代谢物。与其他固相萃取柱相比，MAX固相萃取柱更有助于提高PAEs代谢物的净化效率和回收率。

PAEs代谢物的测定方法主要有气相色谱-质谱法（GC-MS）^［22‒24］^、气相色谱-串联质谱法（GC-MS/MS）^［25］^、液相色谱-质谱法（LC-MS）^［26］^和液相色谱-串联质谱法（LC-MS/MS）^［27］^等。其中，GC需进行衍生化处理，其前处理步骤繁琐且耗时。文献［^28^‒^30^］中报道的液相色谱-质谱联用技术，通常只能分析不超过5种PAEs代谢物，且分析时长均超过10 min。Kato等^［29］^利用在线固相萃取-LC-MS/MS技术，测定了3种PAEs代谢物，但该方法对实验室仪器条件要求较高，仪器参数难以在不同实验室间通用。Myridakis等^［30］^和高慧等^［31］^建立的方法均能够同时测定7种PAEs代谢物，张续等^［32］^建立的方法则能同时测定9种PAEs代谢物，然而这些方法的分析时间至少需要23 min，不利于实现PAEs代谢物的高通量检测。

基于上述问题，本研究将固相萃取与UPLC-MS/MS结合，建立了可同时测定尿液中10种PAEs代谢物的高灵敏度方法。该方法简便、快速，适用于评估大规模人群中PAEs的暴露水平，并能够为筛选和鉴定PAEs暴露的生物标志物提供参考，进而助力全面评估PAEs对人体健康的影响。

## 1 实验部分

### 1.1 仪器、试剂与材料

ACQUITY I-Class超高效液相色谱仪、Xevo TQ-S质谱仪（美国Waters公司）；Master-D超纯水仪（上海和泰仪器有限公司）；5430R冷冻型离心机（德国Eppendorf公司）；KH-200SP超声波清洗机（昆山禾创超声仪器有限公司）；IKA MS 3 digital圆周振荡器（广州艾卡仪器设备有限公司）；Secura 125-1CN分析天平（上海赛多利斯贸易有限公司）。

10种PAEs代谢物标准品和同位素内标均购自加拿大TRC公司。10种PAEs代谢物：邻苯二甲酸单甲酯（MMP，纯度98%）、邻苯二甲酸单乙酯（MEP，纯度98%）、邻苯二甲酸单苯酯（MBzP，纯度98%）、邻苯二甲酸单正丁酯（MnBP，纯度98%）、邻苯二甲酸单（2-乙基-5-氧代己基）酯（MEOHP，纯度97%）、邻苯二甲酸单正辛酯（MnOP，纯度97%）、邻苯二甲酸单异丁酯（MiBP，纯度98%）、邻苯二甲酸单正戊酯（MPP，纯度98%）、邻苯二甲酸单环己酯（MCHP，纯度98%）和环己二甲酸单（4-甲基-7-羧庚基）酯（cx-MINCH，纯度96%），结构式见图1。10种同位素内标：MMP-d4（纯度98%）、MEP-d4（纯度98%）、MBzP-d4（纯度96%）、MnBP-d4（纯度97%）、MEOHP-d4（纯度97%）、MnOP-d4（纯度96%）、MiBP-d4（纯度98%）、MPP-d4（纯度98%）、MCHP-d4（纯度98%）、cx-MINCH-d8（纯度96%）。甲酸（LC-MS级，上海阿拉丁生化科技股份有限公司）；甲醇、乙腈、乙酸铵（LC-MS级，上海安谱实验科技股份有限公司）；*β*-葡萄糖醛酸酶（无锡微色谱生物科技有限公司）；25%氨水（国药集团化学试剂有限公司）。实验中用到的所有超纯水均由Master touch-D超纯水仪制备。Purphy MAX 96孔固相萃取板购自裕菁科技（上海）有限公司。

**图1:**
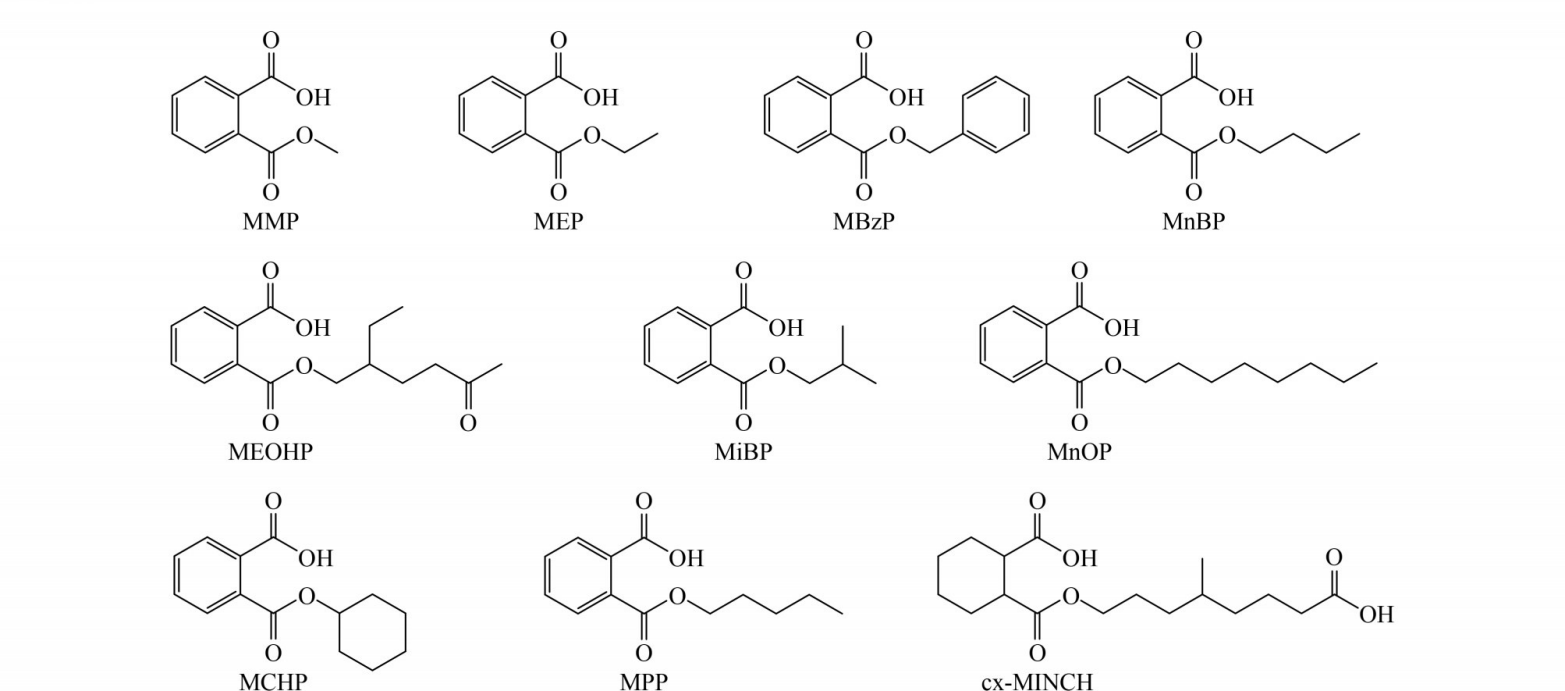
10种PAEs代谢物的结构式

本研究已得到重庆大学附属肿瘤医院伦理委员会的批准（批准文件号：CZLS2022098-A）。尿液采集于表观健康志愿者，且每位志愿者均在试验前签署了知情同意书。

### 1.2 溶液的配制

#### 1.2.1 混合标准溶液和质控工作溶液的配制

精密称取各标准品适量，用甲醇溶解、定容，配制成质量浓度为1 mg/mL的标准储备溶液。精密吸取适量的各标准储备溶液于同一容量瓶中，用甲醇定容，配制成混合储备溶液，其中MMP、MEP、MBzP、MnBP、MnOP和MiBP的质量浓度为100 μg/mL，MEOHP、MPP、MCHP和cx-MINCH的质量浓度为20 μg/mL。用20%甲醇水溶液对混合储备溶液进行逐级稀释，配制成系列质量浓度的混合标准溶液，其中MMP、MEP、MBzP、MnBP、MnOP和MiBP的系列质量浓度为0.05、0.1、0.5、1、2.5、5、10 μg/mL，MEOHP、MPP、MCHP和cx-MINCH的系列质量浓度为0.01、0.02、0.1、0.2、0.5、1、2 μg/mL。随后将配制好的混合标准溶液转移至棕色玻璃瓶中，并于‒80 ℃保存。

采用相同的方法配制各质控储备溶液，混合后用20%甲醇水进行稀释，配制成4个不同水平（定量下限（LLOQ）、低水平质控（LQC）、中水平质控（MQC）和高水平质控（HQC））的质控工作溶液，并储存在棕色玻璃瓶中，‒80 ℃下保存。其中，MMP、MEP、MBzP、MnBP、MnOP、MiBP等6种PAEs代谢物的LLOQ、LQC、MQC和HQC分别为0.05、0.15、2、8 μg/mL；MEOHP、MPP、MCHP、cx-MINCH等4种PAEs代谢物的LLOQ、LQC、MQC和HQC分别0.01、0.03、0.4、1.6 μg/mL。

#### 1.2.2 混合内标工作溶液的配制

精密称取各内标标准品适量，用甲醇溶解、定容，制备内标储备溶液，其中MMP-d4的质量浓度为1 000 μg/mL，MEP-d4、MBzP-d4、MnBP-d4、MnOP-d4、MiBP-d4、MPP-d4、MCHP-d4的质量浓度为500 μg/mL，MEOHP-d4、cx-MINCH-d8的质量浓度为100 μg/mL。用乙酸铵溶液对上述内标储备溶液进行混合稀释，制备混合内标工作溶液，其中MMP-d4、MEP-d4、MBzP-d4、MnBP-d4、MnOP-d4、MiBP-d4的质量浓度为240 ng/mL，MEOHP-d4、MPP-d4、MCHP-d4、cx-MINCH-d8的质量浓度为48 ng/mL。将配制好的混合内标工作溶液储存在棕色玻璃瓶中，并在‒80 ℃下保存。

#### 1.2.3 基质匹配混合标准溶液和基质匹配质控溶液的配制

取11份900 μL空白人工尿液于1.5 mL试管中（编号1~11），向编号为1~7的试管中分别加入1.2.1节系列质量浓度的混合标准溶液（各100 μL），制得10种PAEs代谢物的基质匹配混合标准溶液；向编号为8~11的试管中分别加入1.2.1节4个水平的质控工作溶液（各100 μL），制得10种PAEs代谢物的基质匹配质控溶液。

### 1.3 样品前处理

精密吸取200 μL待测尿液样品于2 mL离心管中，分别加入50 μL混合内标工作溶液和10 μL *β*-葡萄糖醛酸酶溶液，于室温下涡旋混匀1 min，并在55 ℃下酶解1 h；待冷却至室温后，加入100 μL 25%氨水，涡旋混匀1 min，于4 ℃、14 000 r/min下离心3 min，取出上清液，待用。依次用200 μL乙腈和200 μL超纯水活化并平衡Purphy MAX 96孔固相萃取板；吸取300 μL样品上清液，上样于活化后的固相萃取板中，随后依次用200 μL超纯水和200 μL乙腈进行淋洗；更换新的收集板，向固相萃取板中加入50 μL 2%甲酸乙腈溶液进行洗脱，最后向收集板中加入150 μL超纯水，涡旋振荡1 min后，进样分析。

### 1.4 分析条件

#### 1.4.1 色谱条件

色谱柱：ACQUITY UPLC BEH Phenyl柱（50 mm×2.1 mm， 1.7 μm）；流动相：A为0.1%甲酸水，B为0.1%甲酸乙腈；流速：0.5 mL/min；进样量：20 μL；柱温：40 ℃；进样室温度：4 ℃。梯度洗脱程序：0~0.5 min，80% A；0.5~2.5 min，80% A~70% A，2.5~4.0 min，70% A~30% A；4.0~5.0 min，30% A~5% A；5.0~6.0，5% A~80% A。

#### 1.4.2 质谱条件

离子源：电喷雾电离（ESI）源，负离子扫描；数据采集模式：多反应监测（MRM）模式；离子源温度：150 ℃；脱溶剂气温度：550 ℃；毛细管电压：2 kV；脱溶剂气流量：1 100 L/h；锥孔气流量：150 L/h；保留时间和其他质谱参数见表1。

**表 1:** 10种PAEs代谢物及其同位素内标的保留时间和质谱参数

Compound	*t*_R_/min	Parent ion （*m/z）*	Daughter ions （*m/z）*	CV/V	CE/V
MMP	0.90	178.9	107.0^*^， 77.0	20	10， 16
MEP	1.32	193.0	77.1^*^， 121.1	32	14， 12
MiBP	2.76	221.0	134.1^*^， 77.1	42	10， 15
MnBP	2.87	221.0	71.0^*^， 149.1	20	14， 10
MEOHP	3.04	291.1	143.1^*^， 121.1	20	12， 20
MBzP	3.23	255.0	77.1^*^， 104.6	22	18， 16
MCHP	3.34	247.1	97.1^*^， 77.1	12	14， 21
MPP	3.41	235.0	85.1^*^， 77.1	22	14， 18
cx-MINCH	3.45	327.1	173.1^*^， 153	42	16， 24
MnOP	4.04	277.1	127.1^*^， 77.1	20	14， 19
MMP-d4	0.88	183.0	81.0	30	16
MEP-d4	1.31	197.1	81.1	18	14
MiBP-d4	2.75	225.1	138.1	20	14
MnBP-d4	2.85	225.1	81.1	24	20
MEOHP-d4	3.02	295.1	143.1	20	12
MBzP-d4	3.21	259.1	187.1	36	12
MCHP-d4	3.32	251.0	97.0	20	14
MPP-d4	3.40	239.1	81.1	20	16
cx-MINCH-d8	3.44	335.1	173.1	16	14
MnOP-d4	4.02	281.1	127.1	20	18

RT： retention time； CV： cone voltage； CE： collision energy； * quantitative ion.

## 2 结果与讨论

### 2.1 UPLC-MS/MS条件优化

为了提高色谱峰的分离效果和目标化合物的检测灵敏度，需筛选合适的色谱柱和流动相。实验分别考察了ACQUITY UPLC BEH C_8_（50 mm×2.1 mm， 1.7 μm）、ACQUITY UPLC HSS T_3_（50 mm×2.1 mm， 1.8 μm）和ACQUITY UPLC BEH Phenyl（50 mm×2.1 mm， 1.7 μm）3种色谱柱对目标化合物分离效果的影响。结果表明，10种目标化合物在Phenyl柱上的分离效果最好，在6 min内即可实现有效分离且峰形良好。因此，选择Phenyl柱作为分离柱。

实验分别考察了甲醇和乙腈作为流动相有机相时的色谱分离效果。结果表明，当使用甲醇作为有机相时，目标化合物未能得到有效分离，而改用乙腈作为有机相时，色谱峰之间的分离度得到了明显改善。因此，选择乙腈作为流动相的有机相。为了改善色谱峰形，实验进一步考察了水-乙腈、0.1%甲酸水-乙腈和0.1%甲酸水-0.1%甲酸乙腈作为流动相时目标化合物的分离效果。实验结果表明，在0.1%甲酸水-0.1%甲酸乙腈流动相条件下，10种目标化合物的色谱峰无拖尾现象，且响应强度更高。分析其原因，PAEs代谢物是弱酸性物质，在添加甲酸后可能增强了其在反相色谱柱上的保留能力。因此，实验最终选择0.1%甲酸水和0.1%甲酸乙腈作为流动相。

PAEs代谢物属于极性较大的化合物，因此选择ESI^‒^模式进行离子化，并在MRM模式下采集数据。在此基础上，我们进一步优化了质谱参数，包括锥孔电压（CV）、碰撞能量（CE）、离子源温度、脱溶剂气温度以及脱溶剂气流量等，以提升各目标化合物的离子化效率，并最终确定了最优的质谱条件（见表1）。在最佳仪器分析条件下，10种PAEs代谢物的色谱图如图2所示。

**图2:**
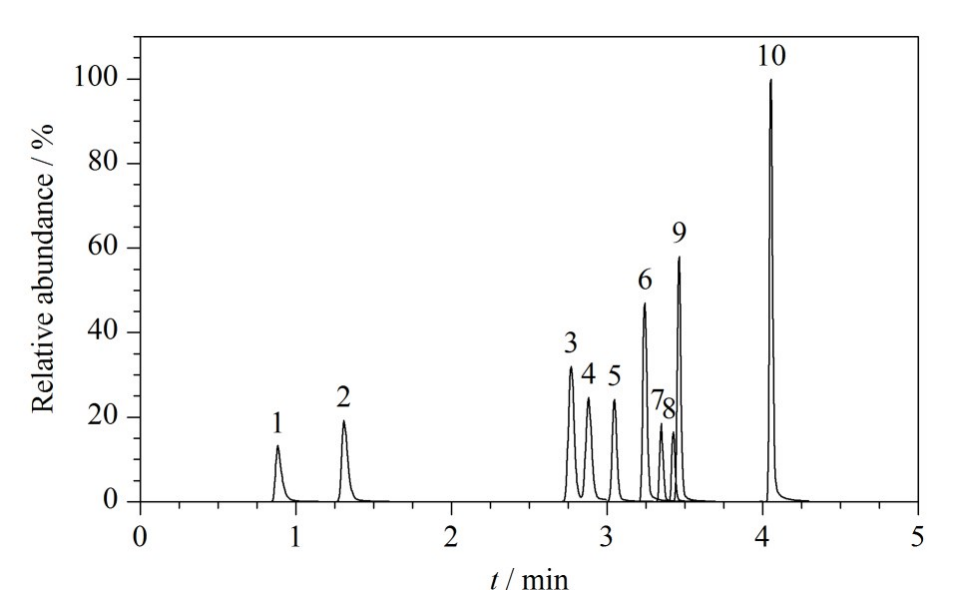
10种PAEs代谢物的MRM色谱图

### 2.2 稳定性考察

按1.2.3节步骤，制备两个水平（LQC和HQC）的基质匹配质控样本，并对这两个样本进行了以下稳定性的考察：（1）在室温下放置5 h后进行前处理；（2）经历3次冻融循环后进行前处理；（3）‒80 ℃下冷冻保存20 d后进行前处理；（4）采样后直接进行前处理，之后在4 ℃的进样器中放置24 h。每个考察条件下的样本平行制备6份，按1.4节条件进样分析，计算相对误差（RE=（目标物含量测定值‒目标物含量理论值）/目标物含量理论值）。实验结果表明，在室温下放置5 h后，10种PAEs代谢物含量的RE为‒7.6%~8.4%；在经过3次冻融循环后，10种PAEs代谢物含量的RE为‒8.3%~6.4%；在‒80 ℃下冷冻保存20 d后，10种PAEs代谢物含量的RE为‒9.5%~0.8%；经前处理并在进样器（4 ℃）中放置24 h后，10种PAEs代谢物含量的RE为‒6.8%~11.3%。实验结果表明，在上述处理条件下，10种PAEs代谢物均能够保持良好的稳定性。

### 2.3 方法学验证

#### 2.3.1 专属性

取200 μL空白人工尿液，按1.3节步骤进行样品前处理，其中混合内标工作溶液用乙酸铵溶液代替，得到不含内标和目标化合物的双空白样本。按1.4节条件进样分析，所得到的色谱图见附图1（www.chrom-China.com）。用200 μL空白人工尿液配制LLOQ水平的基质匹配质控样本，按1.3节步骤进行样品前处理，按1.4节条件进样分析，所得到的色谱图见附图2。实验结果说明，尿液基质不会干扰目标化合物的检测，说明本方法的专属性较高。

#### 2.3.2 线性范围、检出限与定量限

按1.2.3节方法配制系列质量浓度的基质匹配混合标准溶液，其中MMP、MEP、MBzP、MnBP、MnOP和MiBP等6种PAEs代谢物的系列质量浓度分别为5、10、50、100、250、500、1000 ng/mL（相应的内标质量浓度为240 ng/mL），MEOHP、MPP、MCHP、cx-MINCH等4种PAEs代谢物的系列质量浓度分别为1、2、10、20、50、100、200 ng/mL（相应的内标质量浓度为48.0 ng/mL），按照1.4节条件进样分析。以目标化合物的质量浓度为横坐标（*x*，ng/mL），目标化合物与对应内标的定量离子峰面积之比为纵坐标（*y*），采用加权（权重系数为（1/*x* ^2^））最小二乘法绘制标准曲线，并计算回归方程。结果如表2所示，MMP、MEP、MBzP、MnBP、MnOP和MiBP在5~1000 ng/mL范围内线性关系良好，MEOHP、MPP、MCHP和cx-MINCH在1~200 ng/mL范围内线性关系良好，相关系数（*r* ^2^）均≥0.997 1。以3倍信噪比计算检出限（LOD）、10倍信噪比计算定量限（LOQ），10种PAEs代谢物的LOD为0.03~0.3 ng/mL，LOQ为0.1~1 ng/mL。

**表 2:** 10种PAEs代谢物的回归方程、相关系数、检出限和定量限

Compound	Regression equation	*r* ^2^	LOD/（ng/mL）	LOQ/（ng/mL）
MMP	*y*=0.016*x*+0.0074	0.9996	0.3	1
MEP	*y*=0.0195*x*+0.0051	0.9979	0.2	0.5
MiBP	*y*=0.0097*x*+0.005	0.9980	0.3	1
MnBP	*y*=0.0091*x*+0.011	0.9971	0.3	1
MEOHP	*y*=0.0485*x*+0.0017	0.9999	0.03	0.1
MBzP	*y*=0.0078*x*+0.0008	0.9995	0.03	0.1
MCHP	*y*=0.1153*x*‒0.0083	0.9989	0.03	0.1
MPP	*y*=0.093*x*+0.0028	0.9987	0.2	0.5
cx-MINCH	*y*=0.0935*x*‒0.0036	0.9995	0.03	0.1
MnOP	*y*=0.0155*x*‒0.0026	0.9989	0.03	0.1

*y*： peak area ratio of the target compound to the corresponding interal standard； *x*： mass concentration， ng/mL.

#### 2.3.3 残留考察

按1.2.3节和2.3.1节，分别配制系列质量浓度的基质匹配混合标准溶液和不含内标及目标化合物的双空白样本，按1.3节方法进行前处理。随后，从最低质量浓度开始，依次进样系列质量浓度的基质匹配混合标准溶液，在最高质量浓度的溶液进样完成后，立即采集一针双空白样本。结果表明，在双空白样本中，各目标化合物的峰面积占最低质量浓度样本中对应目标化合物峰面积的0~17.3%，均不超过最低质量浓度样本峰面积的20%；同时，双空白样本中各内标的峰面积占最低质量浓度样本中对应内标峰面积的0~0.06%，均不超过最低质量浓度样本内标峰面积的5%。这一实验结果说明，在进样高质量浓度样本后，PAEs代谢物及其内标在系统中产生的残留有限，不会对后续样本的准确测定造成影响，符合生物样品分析的相关规范要求^［33］^。

#### 2.3.4 准确度和日内、日间精密度

按1.2.3节制备4个水平（LLOQ、LQC、MQC、HQC）的基质匹配质控样本，每个水平样本平行制备6份，按1.3节方法进行前处理，按1.4节条件进样分析，计算相对标准偏差（RSD），得到日内精密度（intra-day RSD）；连续进样3 d，计算日间精密度（inter-day RSD）；计算10种PAEs代谢物水平的RE，通过计算3 d内各目标化合物的RE均值来考察方法的准确度。如表3所示，10种PAEs代谢物的日内、日间精密度均不超过8.3%，准确度为‒10.5%~7.3%，符合生物样品分析的相关规范要求^［33］^。

**表 3:** 10种PAEs代谢物在4个质控水平下的准确度和日内、日间精密度

Compound	QC level/（ng/mL）	Intra-day RSD/% （*n*=6）	Inter-day RSD/% （*n*=18）	Accuracy/% （*n*=18）
MMP	5	4.3	4.8	‒0.4
	15	2.4	3.3	3.6
	200	0.7	1.7	3.4
	800	2.3	3.2	1.7
MEP	5	2.8	8.2	‒2.1
	15	1.6	4.9	4.9
	200	1.0	4.2	6.1
	800	1.7	5.4	0.3
MiBP	5	3.6	5.0	2.0
	15	2.1	2.4	4.6
	200	0.3	1.6	5.4
	800	1.4	2.5	‒1.1
MnBP	5	2.2	3.8	‒2.3
	15	2.5	4.3	‒1.0
	200	2.3	3.4	‒2.4
	800	2.0	3.4	‒6.3
MEOHP	1	5.0	4.2	‒1.2
	3	2.6	3.4	3.8
	40	1.5	1.8	3.3
	160	1.4	1.7	0.1
MBzP	5	6.3	7.3	‒3.3
	15	1.5	2.4	4.0
	200	3.2	3.0	4.8
	800	1.4	2.4	0.6
MCHP	1	6.1	8.3	‒6.1
	3	2.9	4.7	1.9
	40	2.5	2.9	0.9
	160	2.3	3.4	0.4
MPP	1	6.6	5.3	‒10.5
	3	5.5	4.9	‒2.2
	40	2.7	3.3	‒1.4
	160	3.6	3.3	0.5
cx-MINCH	1	3.1	3.9	‒4.9
	3	1.6	2.2	1.3
	40	2.6	2.4	0.5
	160	1.5	1.8	‒1.1
MnOP	5	2.9	4.0	1.8
	15	1.3	2.6	7.1
	200	1.4	3.1	7.3
	800	1.7	3.4	7.2

#### 2.3.5 基质效应

尿液基质可能会对目标化合物的准确定量产生干扰，为降低基质效应（matrix effect，ME），本实验采用同位素内标法进行定量。为进一步验证本方法的适用性，考察了尿液对10种PAEs代谢物的基质效应。各取200 μL不同受试者来源（共6位）的实际尿液（每个样本来源平行处理3份），向同一来源的3个样本中分别加入低、中、高3个水平（LQC、MQC、HQC）的质控工作溶液。按1.3节方法进行前处理，随后进样分析，得到目标化合物峰面积与内标峰面积的比值（*A*）；同时，以200 μL超纯水代替实际尿液，按照相同方法操作，随后进样分析，得到目标化合物峰面积与内标峰面积的比值（*B*）。根据ME=*A*/*B*×100%计算基质效应，并计算基质效应的RSD（*n*=6）。结果表明，在3个质控水平下，10种PAEs代谢物的ME为86.5%~113.7%，RSD均不超过15%，符合生物样品分析的相关规范要求^［33］^，不会干扰10种PAEs代谢物的准确测定。

#### 2.3.6 回收率

向空白人工尿液中分别添加低、中、高3个水平（LQC、MQC、HQC）的质控工作溶液，按1.3节方法进行前处理，每个质控水平平行测定6次，并计算回收率和RSD。实验结果表明，10种PAEs代谢物在3个质控水平下的回收率为91.7%~102.9%，RSD为1.0%~6.4%，说明所建分析方法的回收率和精密度均良好，能够满足对实际样本的检验分析要求。

### 2.4 实际样品测定

选择60名长期居住在上海的志愿者参与本研究。收集这些志愿者在早晨5点至8点期间的晨尿样本，随后按1.3节方法进行前处理，按1.4节条件进样分析。实验结果显示，每位志愿者的尿液中均可检出1~6种PAEs代谢物，其中MEOHP、MnBP、MMP和MiBP是较为常见的检出物质，它们的检出率分别为100%、97%、75%和60%；而MBzP、MnOP、MPP和MCHP在60名志愿者的尿液中均未检出。具体结果见表4。

**表 4:** 60名志愿者尿液中10种PAEs代谢物的检出含量

Sample	Contents/（ng/mL）									
	MMP	MEP	MiBP	MnBP	MEOHP	MBzP	MCHP	MPP	cx-MINCH	MnOP
1	14.3	7.6	9.7	28.5	6.2	ND	ND	ND	ND	ND
2	ND	32.0	ND	8.8	60.0	ND	ND	ND	ND	ND
3	ND	ND	ND	25.1	3.2	ND	ND	ND	ND	ND
4	9.4	ND	8.0	48.8	4.2	ND	ND	ND	ND	ND
5	10.7	7.7	14.7	95.5	7.5	ND	ND	ND	ND	ND
6	9.9	16.7	7.8	36.4	1.5	ND	ND	ND	ND	ND
7	14.8	ND	16.3	53.9	6.3	ND	ND	ND	ND	ND
8	5.7	455.9	6.7	34.6	51.7	ND	ND	ND	94.8	ND
9	7.7	44.7	ND	12.1	4.6	ND	ND	ND	ND	ND
10	88.7	8.1	5.5	22.8	5.5	ND	ND	ND	ND	ND
11	ND	48.3	ND	11.9	1.5	ND	ND	ND	ND	ND
12	5.3	101.8	ND	31.7	39.1	ND	ND	ND	208.3	ND
13	10.7	11.7	10.7	95.8	5.5	ND	ND	ND	ND	ND
14	12.4	ND	9.6	49.5	6.2	ND	ND	ND	ND	ND
15	ND	ND	ND	8.6	6.9	ND	ND	ND	ND	ND
16	ND	ND	ND	17.9	3.7	ND	ND	ND	ND	ND
17	10.1	14.2	16.2	55.6	5.9	ND	ND	ND	ND	ND
18	8.8	ND	ND	11.8	1.3	ND	ND	ND	1.5	ND
19	8.6	6.6	ND	7.5	1.9	ND	ND	ND	ND	ND
20	12.3	5.3	8.9	55.9	5.5	ND	ND	ND	ND	ND
21	5.6	69.1	7.4	24.3	3.2	ND	ND	ND	ND	ND
22	9.3	ND	5.5	13.1	2.0	ND	ND	ND	ND	ND
23	10.0	ND	5.4	25.3	6.1	ND	ND	ND	ND	ND
24	ND	133.6	ND	17.3	1.7	ND	ND	ND	ND	ND
25	7.9	ND	12.8	26.8	6.1	ND	ND	ND	ND	ND
26	ND	ND	8.0	27.8	6.7	ND	ND	ND	ND	ND
27	14.1	ND	ND	20.8	6.1	ND	ND	ND	ND	ND
28	ND	1371.1	ND	12.9	3.0	ND	ND	ND	ND	ND
29	ND	ND	ND	22.6	4.1	ND	ND	ND	ND	ND
30	ND	ND	ND	ND	23.7	ND	ND	ND	ND	ND
31	5.9	ND	7.5	27.3	6.2	ND	ND	ND	ND	ND
32	ND	8.9	ND	64.1	3.9	ND	ND	ND	ND	ND
33	9.0	162.4	ND	30.4	3.7	ND	ND	ND	ND	ND
34	8.9	22.2	ND	36.6	3.4	ND	ND	ND	ND	ND
35	7.5	ND	ND	24.6	2.6	ND	ND	ND	ND	ND
36	ND	ND	ND	21.6	3.2	ND	ND	ND	ND	ND
37	5.5	392.4	11.6	39.2	12.8	ND	ND	ND	ND	ND
38	12.8	7.1	180.5	71.2	12.7	ND	ND	ND	ND	ND
39	9.6	ND	11.8	79.7	5.0	ND	ND	ND	ND	ND
40	9.8	ND	7.6	27.5	36.3	ND	ND	ND	ND	ND
41	5.3	ND	ND	22.0	4.0	ND	ND	ND	ND	ND
42	11.9	13.8	7.3	23.7	11.6	ND	ND	ND	ND	ND
43	5.0	ND	7.1	7.5	4.2	ND	ND	ND	ND	ND
44	9.5	ND	7.5	25.9	3.7	ND	ND	ND	ND	ND
45	7.2	11.5	10.6	89.5	6.8	ND	ND	ND	ND	ND
46	22.1	ND	10.4	62.3	4.1	ND	ND	ND	ND	ND
47	5.4	ND	7.7	9.6	4.8	ND	ND	ND	ND	ND
48	13.3	7.2	7.9	56.0	24.4	ND	ND	ND	ND	ND
49	9.3	ND	6.1	63.7	2.4	ND	ND	ND	ND	ND
50	9.4	ND	7.0	23.7	4.0	ND	ND	ND	ND	ND
51	8.4	6.6	21.5	87.3	9.9	ND	ND	ND	ND	ND
52	8.7	8.8	9.9	63.0	3.9	ND	ND	ND	ND	ND
53	ND	ND	ND	39.9	4.9	ND	ND	ND	1.8	ND
54	ND	ND	ND	25.4	1.7	ND	ND	ND	ND	ND
55	ND	ND	ND	ND	101.6	ND	ND	ND	ND	ND
56	17.3	49.1	30.8	91.7	11.6	ND	ND	ND	ND	ND
57	31.6	ND	15.9	15.0	6.8	ND	ND	ND	ND	ND
58	5.5	1087.6	ND	18.3	3.7	ND	ND	ND	ND	ND
59	7.1	ND	5.6	24.6	4.2	ND	ND	ND	ND	ND
60	10.7	ND	6.4	23.4	8.5	ND	ND	ND	ND	ND

ND： not detected.

## 3 结论

本研究将固相萃取与UPLC-MS/MS结合，建立了可同时测定尿液中10种PAEs代谢物的高灵敏度方法。该方法简便、快速，准确度和精密度高，适用于评估PAEs在大规模人群中的暴露水平。未来，可进一步结合暴露组学研究，以评估PAEs对人体健康的潜在风险，为制定相关污染物的使用法规提供科学依据。

## References

[1] Wormuth M， Scheringer M， Vollenweider M， et al . Risk Anal， 2006， 26（3）： 803 16834635 10.1111/j.1539-6924.2006.00770.x

[2] Kim D Y， Chun S H， Jung Y， et al . Int J Environ Res Public Health， 2020， 17（22）： 8582 33227952 10.3390/ijerph17228582 PMC7699231

[3] Xie H， Han W， Xie Q， et al . J Hazard Mater， 2022， 422： 126848 34403943 10.1016/j.jhazmat.2021.126848 PMC8496910

[4] Wittassek M， Koch H M， Angerer J， et al . Mol Nutr Food Res， 2011， 55（1）： 7 20564479 10.1002/mnfr.201000121

[5] Giuliani A， Zuccarini M， Cichelli A， et al . Int J Environ Res Public Health， 2020， 17（16）： 5699 32764471 10.3390/ijerph17165655 PMC7460375

[6] Pagoni A， Arvaniti O S， Kalantzi O I . Environ Res， 2022， 212： 113194 35358548 10.1016/j.envres.2022.113194

[7] Huang C， Zhang Y J， Liu L Y， et al . Sci Total Environ， 2021， 782： 146806 33836381 10.1016/j.scitotenv.2021.146806

[8] Chang W H， Herianto S， Lee C C， et al . Sci Total Environ， 2021， 786： 147371 33965815 10.1016/j.scitotenv.2021.147371

[9] Nguyen V K， Colacino J A， Arnot J A， et al . Environ Int， 2019， 122： 117 30528102 10.1016/j.envint.2018.10.042 PMC6903703

[10] Golestanzadeh M， Riahi R， Kelishadi R . Environ Sci： Processes Impacts， 2020， 22（4）： 873 10.1039/c9em00512a 32091510

[11] Hlisníková H， Petrovičová I， Kolena B， et al . Int J Environ Res Public Health， 2020， 17（18）： 6811 32961939 10.3390/ijerph17186811 PMC7559247

[12] Yi H， Wu H， Zhu W， et al . Front Cell Dev Biol， 2023， 11： 1154923 37560165 10.3389/fcell.2023.1154923 PMC10407402

[13] Jin S， Cui S， Mu X， et al . Environ Sci Pollut Res， 2023， 30（59）： 123770 10.1007/s11356-023-30924-8 37991617

[14] Wu A H， Franke A A， Wilkens L R， et al . Breast Cancer Res， 2021， 23（1）： 23 33823904 10.1186/s13058-021-01419-6 PMC8025373

[15] Dueñas Moreno J， Mora A， Cervantes Avilés P， et al . Environ Int， 2022， 170： 107550 36219908 10.1016/j.envint.2022.107550

[16] Guo Y， Kannan K . Environ Sci Technol， 2011， 45（8）： 3788 21434628 10.1021/es2002106

[17] Tran H T， Nguyen M K， Hoang H G， et al . Chemosphere， 2022， 307： 135989 35988768 10.1016/j.chemosphere.2022.135989 PMC10052775

[18] Tran T M， Kannan K . Arch Environ Contam Toxicol， 2015， 68（3）： 489 25702083 10.1007/s00244-015-0140-0

[19] Koch H M， Ruther M， Schutze A， et al . Int J Hyg Environ Health， 2017， 220（2 Pt A）： 130 27863804 10.1016/j.ijheh.2016.11.003

[20] Haines D A， Saravanabhavan G， Werry K， et al . Int J Hyg Environ Health， 2017， 220（2 Pt A）： 13 27601095 10.1016/j.ijheh.2016.08.002

[21] Cao Z， Lin S， Zhao F， et al . Environ Int， 2021， 146： 106252 33242729 10.1016/j.envint.2020.106252 PMC7828642

[22] Martens F K， Martens M A . Acta Clin Belg， 2002， 57 Suppl 1： 16 11974437

[23] Kim M， Song N R， Choi J H， et al . Sci Total Environ， 2014， 470/471： 1408 23928369 10.1016/j.scitotenv.2013.07.037

[24] Lin X T， Wang X Y， Zhao J Q . Chinese Journal of Chromatography， 2016， 34（5）：528

[25] Bai C， Liu L， Chen S， et al . Environ Res， 2022， 207： 112657 34979126 10.1016/j.envres.2021.112657

[26] Shih C L， Hsu J Y， Tien C P， et al . J Food Drug Anal， 2019， 27（2）： 585 30987730 10.1016/j.jfda.2018.11.002 PMC9296194

[27] Pia Dima A， De Santis L， Verlengia C， et al . Clin Mass Spectrom， 2020， 18： 54 34820526 10.1016/j.clinms.2020.10.002 PMC8601017

[28] Chen M， Tao L， Collins E M， et al . J Chromatogr B， 2012， 904： 73 10.1016/j.jchromb.2012.07.022 PMC3578285 22884473

[29] Kato K， Shoda S， Takahashi M， et al . J Chromatogr B， 2003， 788（2）： 407 10.1016/s1570-0232(03)00041-2 12705982

[30] Myridakis A， Balaska E， Gkaitatzi C， et al . Anal Bioanal Chem， 2015， 407（9）： 2509 25644523 10.1007/s00216-015-8497-5

[31] Gao H， Xu Y Y， Sun L， et al . Chinese Journal of Chromatography， 2015， 33（6）： 622 26536765 10.3724/sp.j.1123.2015.01037

[32] Zhang X， Qiu T， Fu H， et al . Chinese Journal of Chromatography， 2018， 36（9）： 895 30251518 10.3724/SP.J.1123.2018.04002

[33] Chinese Pharmacopoeia Commission . Guidelines for Validation of Quantitative Analysis Methods of Biological Samples. Beijing： China Medical Science Press， 2020： 466

